# Lost in Transcription: Molecular Mechanisms that Control HIV Latency

**DOI:** 10.3390/v5030902

**Published:** 2013-03-21

**Authors:** Ran Taube, Boris Matija Peterlin

**Affiliations:** 1 The Shraga Segal Department of Microbiology Immunology and Genetics, Faculty of Health Sciences, Ben-Gurion University of the Negev, P.O. Box 653, Beer-Sheva, 84105, Israel; 2 Department of Medicine, Microbiology and Immunology, Rosalind Russell Medical Research Center, University of California at San Francisco, San Francisco, CA 94143, USA; E-Mail: matija.peterlin@ucsf.edu; 3 Department of Virology, Haartman Institute, University of Helsinki, 00014 Helsinki, Finland

**Keywords:** HIV latency, transcriptional interference, epigenetic, Tat, positive transcription elongation factor b (P-TEFb).

## Abstract

Highly active antiretroviral therapy (HAART) has limited the replication and spread of the human immunodeficiency virus (HIV). However, despite treatment, HIV infection persists in latently infected reservoirs, and once therapy is interrupted, viral replication rebounds quickly. Extensive efforts are being directed at eliminating these cell reservoirs. This feat can be achieved by reactivating latent HIV while administering drugs that prevent new rounds of infection and allow the immune system to clear the virus. However, current approaches to HIV eradication have not been effective. Moreover, as HIV latency is multifactorial, the significance of each of its molecular mechanisms is still under debate. Among these, transcriptional repression as a result of reduced levels and activity of the positive transcription elongation factor b (P-TEFb: CDK9/cyclin T) plays a significant role. Therefore, increasing levels of P-TEFb expression and activity is an excellent strategy to stimulate viral gene expression. This review summarizes the multiple steps that cause HIV to enter into latency. It positions the interplay between transcriptionally active and inactive host transcriptional activators and their viral partner Tat as valid targets for the development of new strategies to reactivate latent viral gene expression and eradicate HIV.

## 1. Introduction

Since its discovery 30 years ago, the human immunodeficiency virus (HIV) has turned from a highly epidemic threat to a persistent pathogen that co-exists with its host. This progress is mainly due to the administration of effective highly active antiretroviral therapy (HAART) that targets key viral enzymes, which are essential for the replication of HIV [1]. With the introduction of HAART, the spread of the virus has been substantially diminished and viral RNA levels can be reduced to clinically undetected levels in HIV-infected individuals [1]. Looking back at this troublesome period, one can acknowledge the medical achievements in restricting the virus and improving the quality of life and the survival of HIV-infected people worldwide. 

However, in two aspects of the battle against HIV, the medical community has failed to control the infection. First, an effective vaccine that can inhibit viral replication has not yet been developed. Second, viral infected cell reservoirs that appear early in the infection accumulate in sites that resist eradication of the virus by the currently available antiretroviral therapies. As these pools are highly stable, life-long HAART is required, which, in turn, causes severe side effects and promotes the evolution of viral-resistant strains [2].

## 2. Events of Productive HIV Infection

HIV latency is a multifactorial process. However, the importance of each molecular pathway is still under debate. Latency can be defined as a reversible low-productive state of infection, where infected cells retain the capacity to produce new viral particles [3]. For productive infection to occur, target cells need to be activated. In T cells, this feat can be achieved *via* a wide range of stimuli, including T-cell receptor (TCR) and co-receptor ligation by anti-CD3 and anti-CD28 antibodies, cytokines (IL-1β, IL-7, and TNFα) and PKC modulators (PMA or prostratin) [4,5,6,7,8,9,10]. These non-specific stimuli increase levels of required transcription factors and de-compact chromatin, rendering it accessible for initiation and elongation of HIV transcription. For the former, transcription factors, such as TBP, TAFs, Sp1, AP-1, cMyb, GR, C/EBP, Ets-1, LEF-1 and IRF bind to the HIV promoter [2,12,13,14]. For the latter, NFκB and NFAT [11] are recruited to the HIV enhancer. A functional equilibrium between histone acetyl-transferases (HATs; p300/CBP, PCAF and CN5) and histone deacetylases (HDACs) also affect transcription initiation. Basal transcription factors then position RNA polymerase II (RNAPII) on the HIV transcription start site (TSS). This step is followed by the phosphorylation of serine residues at position 5 (S5) in the heptapeptide (YSPTSPS)_52_ repeats of the C-terminal domain (CTD) of RNAPII by TFIIH/Cdk7. However, on most eukaryotic genes, RNAPII quickly pauses close to the TSS. Pausing of RNAPII occurs also on immediate response genes that are required for cells subjected to acute stress signals. These genes regulate a synchronous expression of downstream effectors that alter transcriptional networks and mediate the adaptation of cells to stress [15].

Early nuclear run-on and RNase protection studies in resting cells demonstrated that transcription does not elongate far on the HIV LTR, and that only short, abortive viral transcripts are generated. In part, this strong block is due to the 5’ stem-loop trans-activating response (TAR) RNA structure, which binds tightly to negative transcription elongation (NELF) and to DRB sensitivity inducing (DSIF) factors. After stalling, RNAPII is released and begins to elongate only after the recruitment of positive transcription elongation factor b (P-TEFb) to the viral promoter *via* NFkB, bromo-domain-containing protein 4 (Brd4) in the Mediator, or the super elongation complex (SEC) [16]. In resting cells, this interplay between positive and negative factors silences HIV transcription, as P-TEFb associates mainly with its inactive partners. However, after the synthesis of Tat, RNAPII begins to elongate efficiently and HIV replication resumes [17]. Tat binds to the bulge region of TAR via its arginine-rich motif (ARM), and to cyclin T1 (CycT1) of P-TEFb through its cysteine-rich activation domain [18]. Tat also interacts with HATs, p300/CBP and PCAF. These interactions lead to the recruitment of the chromatin remodeling complex SWI/SNF/BAF, which de-compacts chromatin and facilitates transcription elongation by displacing restrictive nucleosomes [19,20,21,22,23,24]. Stress signals release more P-TEFb from its inhibitory complex to increase its kinase activity and stimulate the proliferation of cells. The main targets of P-TEFb phosphorylation are NELF and DSIF. CDK9 phosphorylates SPT5 of DSIF and the E/RD RNA binding subunit of NELF, removing RD from TAR and converting DSIF into an elongation factor. These steps release RNAPII from pausing and reverse negative effects of NELF and DSIF on transcription elongation [25,26,27]. CDK9 phosphorylates serine residues at position 2 (S2) in the CTD of RNAPII, which assures proper co-transcriptional processing of nascent viral transcripts [18,28,29,30,31,32,33]. 

P-TEFb was isolated initially from fruit flies as a general transcription factor whose kinase activity was inhibited by the ATP analog 5,6-Dichlorobenzimidazole 1-β -D-ribofuranoside (DRB) [34,35]. As Tat transactivation is also inhibited by DRB, P-TEFb was suggested to mediate effects of Tat [36]. Indeed, biochemical analyses identified the Tat associated kinase (TAK) as the cdc2-like cyclin-dependent kinase, PITALRE [37,38,39,40]. Later, PITALRE was identified as CDK9 [30,35,41]. Finally, CycT1 was isolated as the host co-factor that binds to Tat [18]. Genetic data supported these important biochemical breakthroughs. Chromosome 12, which codes for CycT1, was shown to be essential for optimal interactions between Tat and TAR [42,43]. Additionally, a cysteine at positions 261 in the human CycT1, which is a tyrosine in the murine CycT1, was found to be critical for the binding between Tat and CycT1, clarifying the block to Tat transactivation in rodent cells [44]. 

P-TEFb consists of CDK9 and one of three C-type cyclins, CycT1, CycT2a and CycT2b [45]. However, only CycT1 is the cellular co-factor for Tat. In contrast to CycT1:CDK9, levels of these additional P-TEFb complexes do not vary following activation of CD4+ T cells, or macrophage differentiation [46]. Moreover, they are thought to regulate transcription of different subsets of genes [47]. Thus, CycT2 plays a critical role in mouse embryonic stem cells, and its knockout leads to embryonic lethality in mice [48]. It is also expressed at high levels in adult human skeletal muscle cells and plays an important role in their differentiation [49]. Cumulatively, these results suggest that the degree of redundancy in gene regulation by CycT1 or CycT2 is cell type- and tissue-specific. A third cyclin, CycK, was also thought to partner with CDK9, but was demonstrated later to bind to CDK12 and CDK13 to regulate DNA damage response genes [50,51,52,53]. Indeed, S2 phosphorylation depends greatly on CDK12/13 and possibly CDK11. CycK, CDK12 and CDK13 are also highly expressed in pluripotent embryonic stem cells, but not in their differentiated derivatives or tissue-specific stem cells [54]. In contrast, stable interactions between Tat and P-TEFb lessen the role of these other CTD kinases in HIV transcription. 

Along with using HIV as a model for studying transcription elongation [55], reports on the heat shock 70 (Hsp70) promoter also confirmed the importance of RNAPII pausing as a regulatory step of gene expression [56,57]. Later, other host co-activators such as CIITA and c-Myc were found to associate with P-TEFb for the expression of their target genes [58,59]. The regulation of RNAPII pausing has also been linked to later steps of transcription, such as splicing and 3’ end formation [60]. Although mRNA capping occurs during RNAPII pausing and is stimulated by DSIF [61,62,63], P-TEFb also promotes co-transcriptional mRNA processing and mRNA export [64,65,66,67]. CycT1 also interacts with the cellular splicing factor SKIP, which facilitates Tat-mediated transactivation of HIV [68]. In addition, Tat can affect splice site recognition via the ASF/SF-2 alternate splicing complex [69,70]. Cellular mRNA capping proteins Mce1 and Hcm1, which stimulate co-translational capping and stabilization of nascent HIV transcripts, also associate with Tat and increase HIV gene expression [71,72]. Finally, the capping protein binding complex (CBC) can interact with P-TEFb and RNAPII and is required globally for optimal levels of S2 phosphorylation in the CTD of RNAPII. These findings demonstrate a vital role of CBC in connecting pre-mRNA capping to transcription elongation and alternate splicing via P-TEFb [64,65,67]. 

The crystal structure of the Tat:P-TEFb complex reveals that Tat forms extensive contacts with CycT1 and with the T-loop of CDK9 [73]. This structure explains how sequence variation in Tat is tolerated at certain sites. Importantly, Tat also increases the kinase activity of P-TEFb. These findings suggest that the Tat·P-TEFb complex formation could be disrupted, which might inhibit HIV replication [73].

## 3. Mechanisms that Enforce Entrance of HIV into Latency

The infection of HIV results in the integration of many replication-defective proviruses into the host genome. In other activated cells, a fully competent provirus will integrate and replicate efficiently. Often, these cells will produce infectious viral particles until they are eliminated by cytolytic T cells or die from direct cytopathic effects of the virus. Entrance into a transcriptionally silent state will occur when sub-optimally activated infected cells escape the immune surveillance and become quiescent [2,3,10,74,75]. Establishment and persistence of latency will then correlate with low levels of host cell factors such as NFκB and P-TEFb, which impose limitations on rates of transcription. A hallmark of this repression is the absence of Tat, which acts as a master switch for productive HIV transcription. Below are depicted additional pathways that drive the virus into latency and maintain transcriptional repression.

### 3.1. Epigenetic Constraints– Impact on Transcription Initiation

It is now well established that the profile of chromatin around the integrated provirus influences viral transcription. As heterochromatin is more compact and structured than euchromatin, it is repressive for transcription. The compaction of chromatin and its accessibility for transcription factors depend on post-translational modifications of histones and epigenetic marks. These dictate the rates of transcription initiation and ultimately the levels of Tat. The architecture of chromatin is also influenced by the activation state of the infected cell. In a quiescent state, protein modifying enzymes that are recruited favor chromatin condensation. Around the integrated latent provirus, the Nuc-1 restrictive nucleosome becomes the main target for histone modifications. Open chromatin is then characterized by several histone H3 modifications: H3K9Ac, H3K4me, H3K14Ac H3K27me1, H3K36me and H3K79me. In a condensed chromatin state around the HIV LTR, H3K9m2, H3K9m3, H3K27me2 H3K27me3, H3K79me and H4K20me are commonly found and lessen the accessibility of transcription factors to the viral promoter. These changes affect pre-initiation complex (PIC) assembly as well as RNAPII pausing, thus providing multiple blocks to viral gene expression [76,77,78,79]. They also reflect the site of proviral integration into the host genome, which contributes to this transcriptional silencing. Several histone methyltransferases modify histones. SUV39H1 and G9a are involved in heterochromatin formation at the HIV promoter and as a consequence, increase HIV gene silencing [80,81]. G9a inhibits basal and induced HIV gene expression by TNFα or Tat [82]. In addition to the equilibrium between histone acetyltransferases (HATs) and histone deacetylases (HDACs), several other components are also involved in their recruitment to the viral promoter. Ying Yan 1 (YY1) and the late SV-40 (LSF) transcription factors specifically and synergistically repress HIV transcription initiation and viral production via recruitment of HDACs. That HIV can be reactivated by HDAC inhibitors, e.g. Trichostatin A, SAHA (suberoylanilide hydroxamic acid) or Trapoxin [77], also supports a role for HDACs in the establishment of HIV latency [83,84,85]. Other host factors that recruit HDAC-I to the HIV LTR, include p50 homodimers and the transcriptional repressor c-promoter binding factor (CBF-1), which is a key regulator of the notch signaling pathway, Both bind to the NFκB motifs in the HIV enhancer [86,87]. In agreement with the above data, activation of HIV gene transcription via NFκB leads to the displacement of CBF-1 from the LTR as well as the recruitment of HATs and chromatin-remodeling factors. Low levels of basal transcription factors, NFκB and NFAT, further restrict HIV transcription initiation, and ensure that latent proviruses remain transcriptionally silenced for long periods of time [88,89,90,91]. 

**Figure 1:**
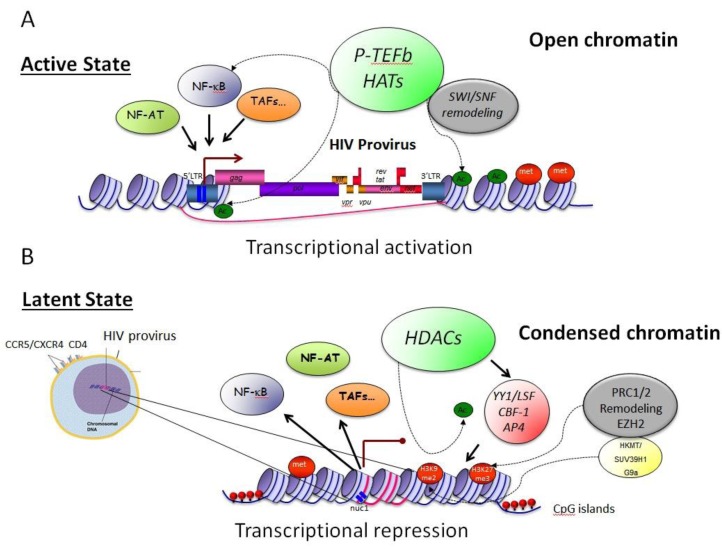
**Epigenetic control modulates HIV latency.** A) In activated T cells, levels of transcription factors (NFκB and NFAT) are elevated, which increases rates of HIV transcription. NFκB (p50/RelA) is tethered to the HIV LTR and recruits P-TEFb, HATs and the SWI/SNF remodeling machinery. This leads to an overall de-compaction of chromatin and higher accessibility for other transcription factors. B) Upon entering the resting state, low levels of transcription factors, NFκB, NFAT and co-activators, P-TEFb, decrease HIV transcription. They also reduce levels of Tat. Epigenetic modifications in the form of de-acetylation of histones as well as methylation of histones and DNA increase the compaction of chromatin and contribute to repression of HIV gene expression. The polycomb repressive complex-2 (PRC2) mediates methylation of histones and DNA, thus inducing gene silencing. HDACs are recruited via p50 homodimers, CBF-1, YY1, AP4, and/or COUP-TF-interacting protein 2 (CTIP2).

In addition to histone methylation, DNA methylation at CpG islands near the HIV promoter has been also correlated with repressive HIV transcription in transformed cell lines but not in primary cells [78,88]. Nevertheless, clearance of HIV from infected patients can be increased by the addition of DNA methylation inhibitors, such as aza-CdR, or NF-κB activators [88,92]. Moreover, the SWI/SNF chromatin remodeling complex BAF, but not PBAF, facilitates the establishment of latency via repressive nucleosome positioning on the HIV LTR [93]. Thus, epigenetic histone modifications and chromatin remodeling machineries, but not DNA methylation, play important roles in HIV latency (Figure 1).

### 3.2. Transcriptional Interference—TI

As mentioned above, for the establishment of latency, HIV integration sites are critical. HIV prefers to integrate into transcriptionally active genes [94,95], where chromatin is relatively open. Less frequently, HIV integrates near centromeric alphoid repeats, or into gene deserts [96,97,98,99,100]. By integrating into active genes, there is a trade-off. While “relaxed” epigenetic patterns around active genes supports integration events and activators assembly on the promoter [78,79], transcriptional interference (TI) between the host and viral promoters displaces transcription factors from the HIV LTR and promotes transcriptional repression. Indeed, RNAPII from the upstream host promoter displaces key transcription factors, like Sp1 and TAFs [97,99,101,102]. In this scenario, ongoing transcription from a host promoter prevents the PIC assembly. Limiting host cell co-activators in the resting state also promote this form of latency. 

In both orientations, the host RNAPII displaces transcription factors and represses HIV transcription at the HIV LTR. Although RNAPII terminates in the 5’ HIV LTR in the sense orientation, in the antisense orientation, it reads through the entire HIV provirus and generates antisense transcripts that are degraded. TI is also a prerequisite for normal viral replication, as HIV needs low affinity transcription factor-binding sites so that it can terminate transcription and polyadenylate viral transcripts at its 3’ HIV LTR. In TI, the host promoter has an advantage: it is not occluded and its affinity for transcription factors can be higher. 

**Figure 2:**
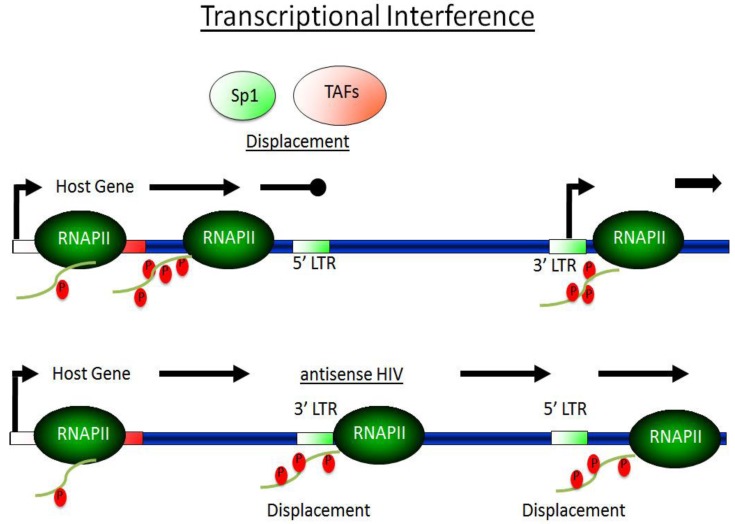
**TI promotes HIV latency**. HIV provirus integrates into actively transcribed genes where chromatin is de-compacted and DNA is accessible to the transcriptional machinery. However, this location also leads to the competition between the integrated viral and host promoters, resulting in transcriptional interference (TI). The provirus integrates in the same or opposite polarity to its host gene. Either way, transcription that initiates from the host gene displaces transcription factors that assemble on the HIV LTR, leading to the silencing of proviral gene expression. In the sense orientation, RNAPII terminates in the 5’ HIV LTR and displaces transcription factors (Sp1/TAFs) (upper panel). In the antisense orientation, transcription factors are displaced from both HIV LTRs; extended antisense HIV transcripts are generated and degraded in infected cells (lower panel).

In intermediate cases where the host gene is not transcribed efficiently, HIV can overcome TI and activate its replication. Given the preferential integrations of viral genomes into active genes [97,99,103], TI is a widespread phenomenon and operates in concert with other mechanisms that enforce latency (Figure 2).

### 3.3 The Dynamic between Transcriptionally Active and Inactive P-TEFb Controls HIV Transcription

Paralleling the regulation of transcription initiation that limits levels of Tat and blocks productive transcription, levels of free active P-TEFb that can bind activators are also tightly regulated in cells. In them, levels of free P-TEFb are restricted, and most of it is found in the large 7SK small nuclear ribonucleoprotein (snRNP). 7SK snRNP consists of the small 7SK nuclear RNA [snRNA], the hexamethylene bisacetamide [HMBA]-inducible protein 1 [HEXIM1], the lupus antigen (La)-related protein 7 [LARP7] and the methylphosphate-capping enzyme [MePCE] [104]. 7SK snRNA acts as a scaffold for the assembly of HEXIM1 and P-TEFb. In this complex, P-TEFb is inactive due to conformational changes of HEXIM1 bound to 7SK snRNA, which blocks the CDK9-ATP binding pocket [105]. The disassembly of the 7SK snRNAP and release of P-TEFb are facilitated by activation and stress signals like apoptosis, UV light, actinomycin D and P-TEFb kinase inhibitors. Acetylation of CycT1 also liberates P-TEFb from its inactive complex [106]. In addition, Tat and the host Brd4 can extract P-TEFb from the 7SK snRNP. Brd4 also associates with acetylated histones via its bromodomains [107]. 

**Figure 3:**
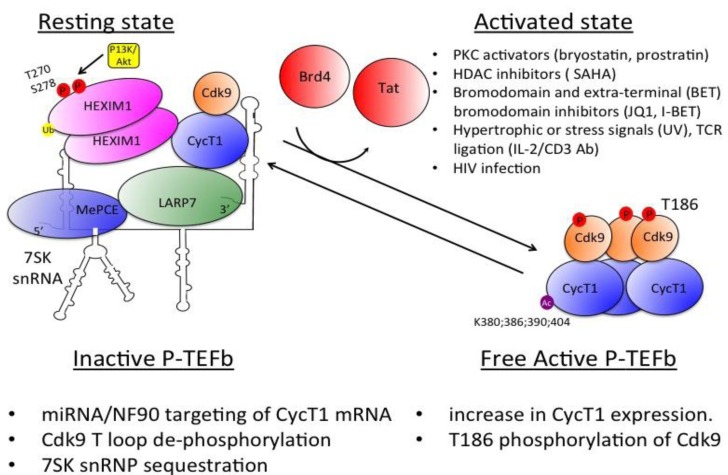
**Interplay between positive and negative complexes regulates P-TEFb transcriptional activity**. In resting cells, the binding of CycT1 to HEXIM1 in the 7SK snRNP inactivates the kinase activity of P-TEFb. In conjunction with low expression levels of P-TEFb and basal transcription factors, transcription is repressed. Activation of CD4+ T-cells or monocytes increases the expression and kinase activity of P-TEFb. Indicated stress signals release P-TEFb from its inactive complex and subsequently lead to its recruitment to the HIV LTR as an active complex, which stimulates transcription elongation. Low levels of specific miRNA that target CycT1 also contribute to P-TEFb activation and cell proliferation. By releasing Brd4 from chromatin, I-BET or JQ1 liberate P-TEFb from the 7SK snRNP and stimulate HIV transcription.

The P-TEFb interacting domain (PID), which is located at the C-terminus of Brd4, is essential for its binding to P-TEFb [108,109,110]. However, almost all Brd4 is associated with interphase chromatin in untreated cells. Upon stress, Brd4 is released from acetylated chromatin via histone deacetylation, and this step is essential for the recruitment of active P-TEFb to promoters and for transcription elongation [111,112]. The second bromodomain motif (BDII) of Brd4 also binds to the acetylated CycT1, thus, interactions between CycT1 and Brd4-tethered chromatin could be mutually exclusive [113]. How P-TEFb-Brd4 complexes transit to and from chromatin in response to external stimuli is still not well understood. The accepted model argues that, following stimulation, the release of P-TEFb from the inactive 7SK snRNP and from chromatin is triggered, thereby allowing Brd4 in the Mediator to recruit P-TEFb to the promoter [114] (Figure 3).

Brd4 and Tat also accelerate the dynamics of mRNA synthesis by supporting chromatin de-compaction and inducing gene activation [115]. Other signals that will be discussed below also lead to T-cell activation and disrupt the 7SK snRNP [46]. These activation signals ultimately alter chromatin structure around the integrated provirus and de-compact it, thus stimulating transcription via basal and Tat-dependent mechanisms [116,117,118,119] (Figure 3).

### 3.4 Regulation of P-TEFb Expression and Activity

Levels of P-TEFb subunits are low in resting CD4+ T cells and monocytes [120,121], but are dramatically elevated upon activation [46,122,123,124]. Increased expression levels also occur upon differentiation of monocytes to macrophages. In macrophages, proteasome-mediated proteolysis of CycT1 limits the expression of CycT1 *via* its C-terminal PEST sequence [125,126]. In addition, in resting monocytes and CD4+ T cells, miR198 and miR27b, 29b, 150 and 223 repress CycT1 expression, respectively [121,127,128]. Nuclear factor 90 (NF-90) also binds to the 3’-UTR of CycT1 and regulates its expression [129]. 

Other post-translational modifications also affect P-TEFb activity. For example, the C-terminal region of CDK9 is auto-phosphorylated and mediates binding to other transcription factors like Tat SF1 and RNAPII and perhaps additional components of the elongation apparatus [130,131]. The un-phosphorylated CDK9 and the C-terminus of CycT1, which folds back to interact with its N-terminus also inhibit the TRM in CycT1. Relief of this auto-inhibition in CycT1 involves a conformational change in CycT1, which unmasks critical TRM sequences and requires the auto-phosphorylation of CDK9 (see below) [18,131,132,133]. Lysine residues located in the coil–coil region of CycT1 are acetylated and mediate association of P-TEFb with Brd4, which can extract P-TEFb from the 7SK snRNP [106,134]. 

CDK9 expression levels and activity are also tightly regulated and depend on the activation and differentiation state of the cell, based on the cell’s metabolic demands [135,136]. In cells, there exists two isoforms of CDK9, which measure 42 kDa and 55 kDa. They differ in their N-terminal region and are transcribed from different promoters. These two isoforms share different sub–cellular localizations and expression patterns. Immunofluorescence studies revealed that whereas the 42-kDa variant of CDK9 is present in nuclear speckles [137,138], the 55-kDa isoform is localized to the nucleolus [139]. Moreover, total CDK9 expression levels are elevated upon cell activation, mainly due to an increase of the 42 KDa isoform [139]. Similarly, the kinase activity of CDK9 is also limited in resting primary cells. 

Basal T-loop (amino acids 168–197) phosphorylation of CDK9 is extremely low in resting CD4+ T-cells [122,123], which further limits P-TEFb activity. Following T-cells activation and CycT1 induction, T-loop phosphorylation increases rapidly. CDK9 is also phosphorylated at other residues (T29, S90, T186, S175, S347, T362, T363) [132,140,141,142,143,144]. These phosphorylation events induce a conformational change of the T loop, allowing entry of the substrate and ATP into the CDK9 catalytic pocket [145]. Thus, the T186 phosphorylation in the conserved T-loop of CDK9 is the critical event for optimal CDK9 activity and association with the 7SK snRNP [140,144]. At present, this kinase remains poorly characterized. Some reports suggest that CDK9 itself can phosphorylate Thr186, which is influenced by Tat or TFIIH [130,131,146]. This situation would be analogous to the auto-phosphorylation of the C terminus of CDK9 on S347, T350, S353, T354 and S357 that also facilitates the assembly of P-TEFb onto TAR [131]. However, the kinase activity of a purified P-TEFb inefficiently auto-phosphorylates T186 *in vitro*, pointing to the involvement of another kinase [110,132,143]. To this end, CDK2 and CDK7 were also identified as potential CDK9 kinases [147,148]. T186-phosphorylated CDK9 is mainly found within the inactive P-TEFb complex [110]. Accordingly, a de-phosphorylation step of CDK9 is important to recycle P-TEFb and regulates its dissociation from the 7SK snRNP [140]. Several phosphatases affect this phosphorylation state of CDK9. The calcium-sensitive and calmodulin-activated serine/threonine phosphatase PP2B and the alpha subunit of protein phosphatase 1 (PP1α) as well as manganese- or magnesium-dependent protein phosphatase 1A and 1B (PPM1A and PPM1B) affect the phosphorylation of T186. PP1α and PP2B, cooperatively release P-TEFb from the 7SK snRNP [110,149,150]. In this scenario, PP2B induces conformational changes in P-TEFb that allow for subsequent dephosphorylation of T186 by PP1α. PPM1A also associates with CDK9 and can de-phosphorylate it regardless of its association with 7SK snRNA. PPM1B does so only when 7SK snRNP is depleted [151]. CDK9 is also poly-ubiquitylated, which targets it for proteolysis via the SCF (Skp1-Cul1-F-box protein) complex, where CycT1 binds to SKP2 [141,152]. Similarly to CycT1, CDK9 is acetylated, presumably on K40 and K44 residues. Since an acetylation-defective mutant CDK9 (K44R) and CDK9 isolated from cells over-expressing HDAC1 or HDAC3 are kinase deficient, the acetylation of CDK9 might be important for its kinase activity. p300 or members of the GCN5-related N-acetyltransferase (GNAT) family (GCN5 and PCAF) mediate this CDK9 acetylation. These effects are direct and impact the kinase activity of CDK9 [142,153].

### 3.5 SEC Associates with HIV Tat and Activates Viral Transcription

**Figure 4:**
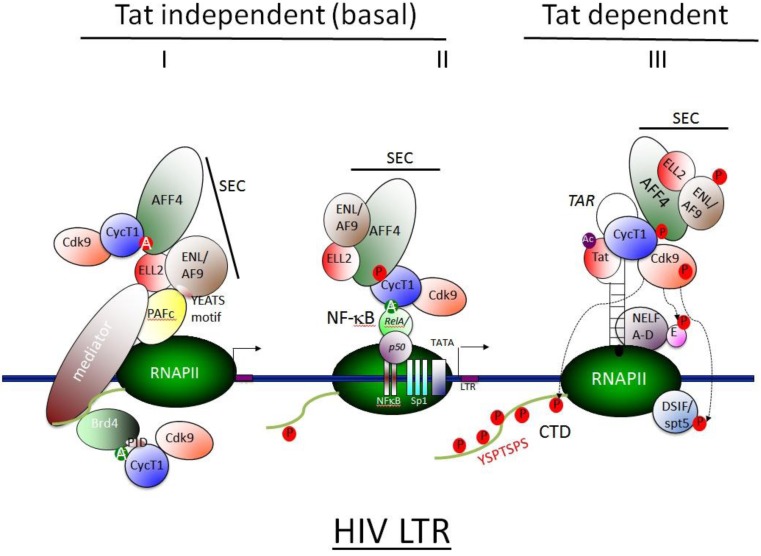
**Recruitment mechanisms of P-TEFb to promoter**. The efficient transcription of signal-inducible genes relies on the release of P-TEFb from its inactive complex and also on its recruitment to promoters. Recruitment of P-TEFb to viral and host promoters leads to stimulation of transcription, thus releasing RNAPII from pausing. Several pathways exist for the recruitment of P-TEFb to the HIV LTR. Among them, Tat-independent basal transcription includes recruitment via NFκB, Brd4 and SEC in the Mediator. Elongation of transcription is enhanced by Tat, which binds P-TEFb and tethers it to TAR. **(i)**Basal transcription from the HIV LTR is maintained by P-TEFb that is recruited to target genes via Brd4 and SEC in the Mediator, which is part of the RNAPII holoenzyme. Herein, Med26 or Cdk8 tether SEC to the Mediator. Within SEC, AFF4 binds to CycT1 and acts as a scaffold that connects P-TEFb to ELL2, which also stimulates transcription. In ENL/AF9 of SEC, the YEATS motif binds RNAPII-associated factor 1 (PAF1) complex and is also recruited to RNAPII in chromatin. P-TEFb may be also recruited to the promoter via Brd4 in the Mediator. This interaction involves tri-acetylated CycT1 and is mediated by the P-TEFb interacting domain (PID) in the C-terminal region of Brd4 and the second bromodomain in Brd4 (BDII). Additionally, the BDII domain of Brd4 associates with acetylated chromatin. However, this interaction does not include active P-TEFb. **(ii)** P-TEFb is also recruited to the HIV promoter in a Tat-independent mechanism. NFκB binds to DNA, tethers CycT1 to the LTR and increases rates of initiation and elongation of transcription. SEC binds to P-TEFb and is in the same complex.

P-TEFb is not the only complex that is recruited by Tat to the viral promoter. In a search of other Tat co-factors, biochemical affinity-purification strategies identified a SEC that consisted of ELL2, AFF4, ENL, AF9 in addition to Tat and P-TEFb [154,155,156]. ELL1 and ELL2 are well-characterized transcription elongation factors that stimulate the activity of RNAPII by keeping the 3**’**-OH of nascent mRNA in alignment with the catalytic site and preventing RNAPII backtracking [157,158,159]. Tat stabilizes ELL2 levels and promotes SEC formation. Despite being in a same complex with Tat, SEC also recruits P-TEFb to the vicinity of RNAPII and increases basal transcription in the absence of Tat. Indeed, SEC interacts efficiently with RNAPII via the Mediator and/or the human polymerase-associated factor complex (PAFc) transcription elongation complexes [160] (Figure 4).

## 4. Therapeutic Approaches

A stable latent reservoir of HIV in resting memory CD4+ T cells is a major barrier for complete viral eradication [2,161,162,163]. Efforts to purge latent HIV have initially focused on reactivating latent proviruses with IL-2 alone or in combination with anti-CD3 antibodies. However, these strategies resulted in severe side effects and had low efficacy. Improved tools should induce HIV transcription without activating cells of the immune system [164,165,166]. In tissue culture, T-cell activation agents like IL-7 [167,168], PKC modulators (phorbol esters (phorbol-12-myristate 13-acetate, PMA), prostratin or bryostatin-1 [169,170]), disulfiram [bis(diethylthiocarbamoyl) disulfide], which inhibits aldehyde dehydrogenase [171, 172], hexamethylene bisacetamide (HMBA), which induces terminal differentiation and apoptosis in transformed cells in culture [173,174,175,176], and HDAC inhibitors valproic acid and SAHA [177,178,179] have all been tested. These compounds stimulate HIV replication by inducing P-TEFb activity and promoting changes in chromatin. Other pathways like recruitment of active NFκB, NFAT and other transcription factors to the viral promoter are also essential for viral reactivation. Clinical studies with valproic acid suggested that they decrease levels of latent viral reservoirs [177,178,179,180,181]. Suberoylanilide hydroxamic acid (SAHA; vorinostat), which has been approved for the treatment of cutaneous T cell lymphoma, releases P-TEFb from the 7SK snRNP and activates HIV transcription [182,183]. Vorinostat could become a component of the “shock and kill” approach, where a “shock” phase reactivates latent proviruses then a “kill” step limits viral replication and spread with HAART [183]. The assumption is that, following the reactivation of the virus, HIV-infected cells will die as a result of host immune responses and/or viral cytopathic effects [184]. However, recent experiments with SAHA in infected patients treated with HAART revealed inadequate levels of CD8+ T-cell-mediated cytotoxic killing of reactivated cells. It is possible that higher concentrations of SAHA, possibly with the combination of other compounds, could lead to higher levels of HIV protein expression, thus higher cytopathic effects of the virus. Alternatively, boosting anti-HIV CTLs via vaccination prior to reactivating latent proviruses may be required for HIV eradication [3].

Initiation events facilitate the accumulation of Tat, which binds to CycT1 and TAR to recruit P-TEFb to the HIV LTR. In a Tat-dependent pathway, P-TEFb mainly supports transcription elongation by phosphorylating subunits of NELF and DSIF to release RNAPII from its pausing on the HIV promoter. P-TEFb also phosphorylates S2 in RNAPII CTD. Tat also associates with the SEC.

The use of compounds that reactivate HIV must occur in the presence of HAART, which will prevent re-infection by replication-competent viruses. Inhibitors of the BET bromodomain - JQ1(S) and I-BET (bromodomain and extraterminal (BET) proteins) - which are in clinical studies for the treatment of several types of cancers including multiple myeloma, also activate HIV gene transcription. They bind to Brd4 (and other members of this family of structural proteins) and displace it from acetylated chromatin and the viral promoter. As a consequence, P-TEFb is released from the 7SK snRNP. Since other members of the BRD family also play important roles, these studies point to new targets for BET bromodomain inhibition in HIV infection [185,186]. 

Overall, an intelligent therapy will likely include a combinatorial approach that will change chromatin, increase the synthesis of P-TEFb as well as release it from the 7SK snRNP. Since P-TEFb levels are extremely low in primary infected resting hematopoietic cells, [120,121], PKC and TLR agonists must first increase their levels. Only then can HDAC or BET bromodomain inhibitors be applied and become effective. These compounds can be administrated, together or sequentially, to relax chromatin and release P-TEFb from its inactive complex. Indeed, little effect of HDACis alone has been reported in primary resting CD4+ T-cells [86,187,188], presumably because levels of P-TEFb are so low. PKC agonists will also induce NFκB, thus stimulating transcription initiation and NFκB-mediated recruitment of P-TEFb. Lower doses of these agonists for longer cycling periods could lead to a functional cure, which will allow the immune system to keep the virus in check. These manipulations will ultimately cause cells to initiate DNA stress responses and avoid death via NFκB and P-TEFb. The release of P-TEFb increases the synthesis of HEXIM1, which restores the P-TEFb equilibrium that not only prevents cell activation and proliferation but hastens the demise of HIV-infected cells.

## 5. Conclusions

To date, only HAART and stem cell transplantation from a CCR5-deleted donor cleared HIV from an infected adult [189]. Thus, the complex network that maintains HIV latency represents a major obstacle to the eradication of the virus. Currently tested drugs that successfully reactivate HIV are still not proven to be effective. At the same time, HAART cannot block ongoing viral replication especially in privileged anatomical sites. Cell-to-cell viral spread also contributes to drug insensitivity and hampers complete suppression of HIV replication. 

Nevertheless, new insights into pathways that drive HIV into latency, as well as the understanding of the molecular mechanisms by which HDAC or BET bromodomain inhibitors activate latent HIV are essential for the development of new therapeutic approaches to the eradication of HIV. We understand today that a combinatorial or synergistic approach will have to take place, as PKC agonists, P-TEFb disruptors and chromatin stress modulators are all partially effective. Issues of drug penetration into tissues also need to be addressed, as viral reservoirs tend to accumulate in poorly accessible sites.
